# Recurrent Respiratory Infections in Children with Down Syndrome: A Review

**DOI:** 10.3390/children11020246

**Published:** 2024-02-15

**Authors:** Michele Ghezzi, Nicolò Garancini, Raffaella De Santis, Laura Gianolio, Salvatore Zirpoli, Anna Mandelli, Andrea Farolfi, Enza D’Auria, Gian Vincenzo Zuccotti

**Affiliations:** 1 Pediatric Department, “Vittore Buzzi” Children’s Hospital, 20154 Milan, Italy; nicolo.garancini@unimi.it (N.G.); raffaella.desantis@unimi.it (R.D.S.); laura.gianolio@unimi.it (L.G.); andrea@farolfi.it (A.F.); enza.dauria@unimi.it (E.D.); gianvincenzo.zuccotti@unimi.it (G.V.Z.); 2 Pediatric Radiology Unit, “Vittore Buzzi” Children’s Hospital, 20154 Milan, Italy; salvatore.zirpoli@asst-fbf-sacco.it; 3 Division of Pediatric Anesthesia and Intensive Care Unit, Department of Pediatrics, “Vittore Buzzi” Children’s Hospital, 20154 Milan, Italy; anna.mandelli@asst-fbf-sacco.it; 4 Department of Biomedical and Clinical Science, Università Degli Studi di Milano, 20157 Milan, Italy

**Keywords:** Down Syndrome, recurrent respiratory infections, tracheomalacia, airway malformations, children, review

## Abstract

Down Syndrome (DS) is the most common chromosomal abnormality compatible with life. The life of patients suffering from DS can be strongly impacted by Recurrent Respiratory tract Infections (RRIs), leading to an increased rate of hospitalisation, a higher need for intensive care and fatality. With a literature review, we summarise here the main etiological factors for RRI in this category of patients, particularly focusing on airway malformations such as tracheomalacia, tracheal bronchus and bronchomalacia, comorbidities associated with the syndrome, like congenital heart diseases, dysphagia, gastroesophageal reflux, musculoskeletal involvement and obesity, and immunologic impairments, involving both innate and adaptive immunity. For these patients, a multidisciplinary approach is imperative as well as some preventive strategies, in particular vaccinations in accordance with their national schedule for immunization.

## 1. Introduction

Down Syndrome (DS) is the most common chromosomal abnormality compatible with life, with a worldwide incidence of approximately 1 in 700 live births annually [1]. DS is characterised by variable cognitive impairment, dysmorphic features and congenital malformations involving several organ systems (mostly cardio-vascular, gastrointestinal tract and respiratory system) [2]. Moreover, children with DS are more vulnerable to upper and lower respiratory tract infections and Recurrent Respiratory tract Infections (RRIs) can strongly impact their life, leading to a higher rate of hospitalisation, increased need for intensive care and a higher chance of fatality [3]. Lastly, even apparently mild, Lower Respiratory Tract Infections (LRTIs) may be associated with lower neurodevelopmental scores compared to the general paediatric population [4].

Multiple factors should be accounted for RRI prevalence and severity: first, airway malformations, such as laryngomalacia, tracheomalacia and lower airway anomalies [5]; second, other comorbidities associated with the syndrome, such as congenital heart diseases, pulmonary hypertension, gastroesophageal reflux disease and hypotonia leading to swallowing dysfunction; third, immunologic impairments, as DS is the genetic syndrome mostly associated with immune defects [6].

The aim of this review is then to briefly describe how different etiological factors, particularly when combined with each other, can lead to RRI in this specific population, aware of the impact that this condition can have on the quality of life of these children.

## 2. Materials and Methods

An extensive literature review was conducted using the search engine PubMed, considering articles published from 1 January 2000. Previous significant publications on the subject have been considered as well. The bibliographic search has been performed using the following keywords: “Down Syndrome”, “Recurrent Respiratory tract Infections”, “Airway malformations”, “Comorbidities” and “Immunologic impairment”. The screening phase has been conducted by three different researchers: only studies with unanimous consensus were included in the review.

## 3. Epidemiology

Currently, the available literature shows that children with Down Syndrome suffer from more frequent infections compared to the general paediatric population; in particular, the lifespan prevalence of pneumonia and respiratory infections is about 20–36% [7,8], with a rate of recurrent pneumonia up to 16–21% [7,9]. In addition, pneumonia represents the most common cause of acute hospital admission in DS children (26% of the total number of hospitalizations [10]) and LRTIs account for 40% of admissions [11], in contrast to the most frequent causes in other children, such as asthma, chemotherapy, fractures, gastroenteritis, bronchiolitis and adenotonsillectomy.

In DS children, it is also clear how these infections can present a more severe clinical course; in particular, pneumonia causes in those patients an increased rate of hospitalisation compared to controls (25.6% of 6869 vs. 6.4% of 99,305, *p* < 0.01) [10] and respiratory infections show a greater likelihood for hospital admission (OR: 2.1, 95% [12]), more often requiring access to a Paediatric Intensive Care Unit (PICU) and mechanical ventilation [11]. Pneumonia is the main diagnosis of admission to the PICU and it is associated with a greater fatality rate when it requires intensive care [13]. This can be explained by the extremely high prevalence of congenital airway anomalies in patients with Down Syndrome and respiratory symptoms, such as chronic cough, recurrent infections and inspiratory stridor (71%, compared with only 32% of controls with similar complaints) [14].

Focusing on pathogens responsible for lower respiratory tract infections, Respiratory Syncytial Virus (RSV) is one of the most common found in young children and DS represents an independent risk factor of severe RSV-associated LRTIs [15]. Children with Down Syndrome are more often hospitalised due to RSV infection and 10% of hospitalised children require mechanical ventilation. A recent retrospective case series (*Löwensteyn and al., Respiratory Syncytial Virus-Related Death in Children with Down Syndrome: The RSV GOLD Study*) estimates that one quarter of children with DS and RSV-confirmed death did not have other risk factors such as prematurity, congenital heart disease and chronic lung disease [16]. The relative risk for hospital admission is 6- to 8-fold higher and the relative risk of mortality is approximately 9-fold higher compared with children without DS [17]. For all these reasons, this population needs protection against RSV infection; currently, the only strategy is passive immunisation by RSV monoclonal antibodies (mainly palivizumab, the new nirsevimab is still not approved for the daily clinical practice in every country) but guidelines regarding its use in children with DS are still controversial.

Influenza is another frequent respiratory infection worldwide: although recent studies suggested that it may be less frequent among people with Down Syndrome [18], influenza infections and pneumonia have a significantly higher mortality rate in all age groups in patients with Trisomy 21 than in controls (AOR 9.93) [19].

To summarise, pneumonia is widely seen as an important cause of morbidity in childhood and of mortality especially in adults with DS, mainly due to respiratory failure [20].

## 4. Aetiology

The main factors responsible for recurrent respiratory infections in DS children are analysed below, divided into three main categories: airway malformations, comorbidities associated with the syndrome and immunologic impairments; those are summarised in Table 1.

### 4.1. Airway Malformations

Airway malformations associated with DS may be responsible for morbidity and mortality in children. The upper airway may be often obstructed in this population due to several phenotypic features (e.g., macroglossia, midface hypoplasia, narrow nasopharynx) and other associated conditions (e.g., adenotonsillar hypertrophy, choanal stenosis, obesity) [21].

Conditions affecting the larynx and trachea are also common. Laryngomalacia, a congenital softening of the laryngeal tissues above the vocal cords causing stridor, is the most common finding in infants and children with Down Syndrome in the first two years of life [22], with a prevalence of approximately 50% [5]. This condition is also linked to gastro-oesophageal reflux disease and hypotonia.

Subglottic stenosis, defined as a congenital or acquired (e.g., after prolonged intubation) narrowing of at least half of the subglottic larynx, is a more common airway anomaly in DS children compared to the general paediatric population (prevalence of 6% vs. 0.6%). It can also be a cause of Obstructive Sleep Apnoea (OSA) [23]. In DS children, the trachea is usually narrower with the presence of complete tracheal rings (tracheal stenosis), forcing the clinicians to the use of smaller endotracheal tubes when needed.

**Figure 1:**
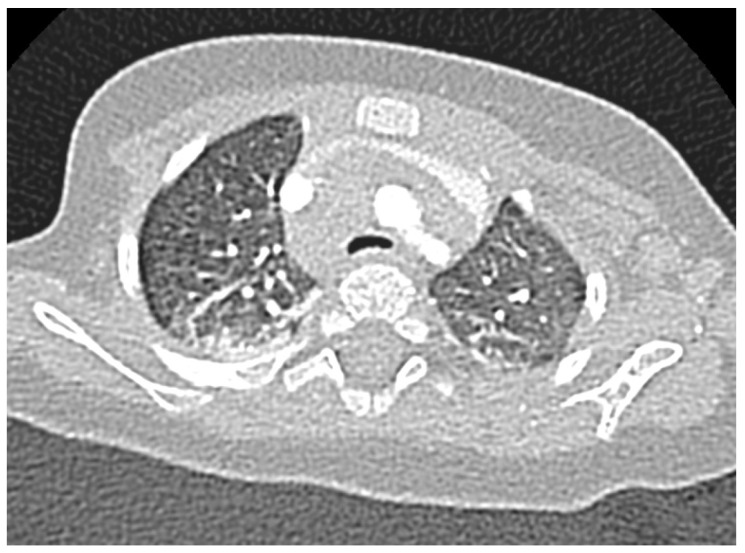
Tracheomalacia in a child with Down Syndrome, axial section.

**Figure 2:**
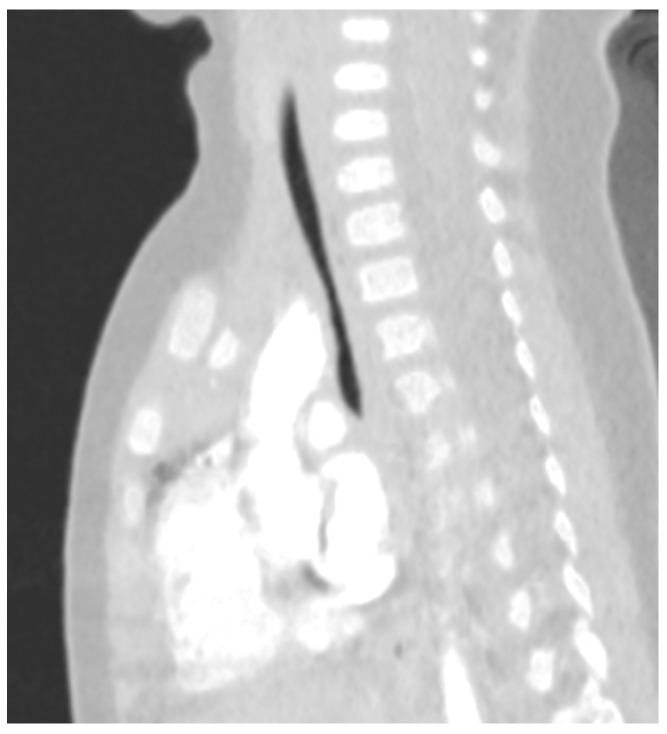
Tracheomalacia in a child with Down Syndrome, sagittal section.

Lastly, tracheomalacia, a condition where the cross-sectional area of the airway can collapse more than 50% during quiet breathing, is the result of cartilage ring malformation or localised softening [24]; this condition is also more common in the DS population with a prevalence of 33% vs. 7.4% [25]. Most cases improve spontaneously with growth but intervention may be needed in significantly symptomatic children, with conservative treatment (pharmacological therapy, chest physiotherapy and respiratory support, mainly with CPAP) or surgical intervention (e.g., anterior or posterior tracheopexy) [26]. In Figure 1 and Figure 2, a narrowing of the antero-posterior cross-sectional area of the trachea is shown, consistent with a diagnosis of tracheomalacia.

**Figure 3:**
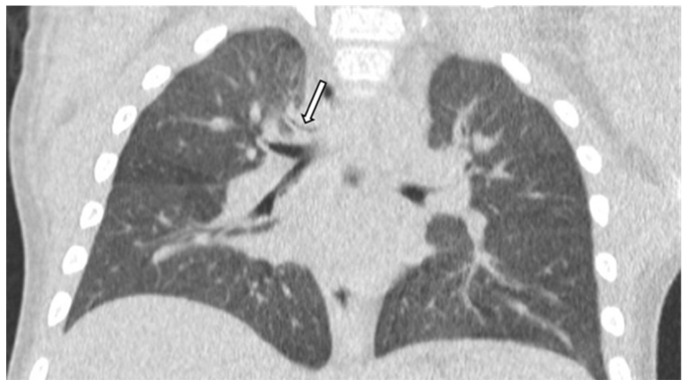
Tracheal bronchus, indicated by the arrow, in a child with Down Syndrome.

Bronchomalacia, which has criteria similar to those for tracheomalacia, and tracheal bronchus, which can be defined as an ectopic bronchus arising from the lateral wall of the trachea above the main carina and supplying the right upper lobe, are also more common in the DS population rather than among the controls (respectively, 7.7% vs. 4% and 3.1% vs. 1.3%), though with a smaller significance level [14]. Tracheal bronchus seems to have a relevant association with respiratory symptoms, particularly obstructive bronchitis and recurrent pneumonia, usually affecting the right upper lobe. It is often associated with other congenital anomalies, even if it is not possible to determine its exact prevalence in the general population and in asymptomatic children. In Figure 3, a tracheal bronchus is shown, arising from the right lateral wall of the trachea, in a DS child.

The diagnosis of tracheomalacia, bronchomalacia and tracheal bronchus is usually made by bronchoscopy, which represents the gold standard technique, but a dynamic chest CT scan could provide other important information about the possible presence of vascular rings or slings or about the presence of complications, such as bronchiectasis to complete the diagnostic work-up [27]. MRI is also increasingly used in order to reduce potentially harmful effects of radiations. Thanks to recent innovations, it is now possible to address some major obstacles unique to the thorax, such as the motion artifacts related to breathing or the low signal-to-noise ratio linked to the air in the chest that had prevented its spread; for example, an MRI-compatible spirometer can allow the respiratory cycle to coordinate with the image acquisition [28].

In conclusion, it is also a remarkable finding that multiple airway malformations are present in 20% of the DS population vs. 5% of controls [14].

### 4.2. Comorbidities Associated

DS is associated with over 80 conditions such as congenital anomalies, sleep breathing disturbances, neurodevelopmental disorders and autoimmune diseases [29]. Those additional diagnoses often pose an increased risk of pneumonia, specifically in children who had undergone orthopaedic, intestinal or airway procedures or were undergoing treatment for DS-Acute Myeloid Leukaemia [3].

#### 4.2.1. Cardiovascular Involvement

Cardiovascular disease is a leading cause of morbidity and mortality in children with DS. Congenital Heart Diseases (CHDs, present in up to 50% of people with DS), such as atrioventricular septal defects and ventricular septal defects but also tetralogy of Fallot, contribute to poor outcomes, especially when associated with Pulmonary Hypertension (PH) [30]. The link between PH and respiratory disease is biunivocal: pulmonary hypertension, which is typically transient, can recur in the setting of respiratory disease such as OSA, intermittent hypoxia and recurrent pneumonia but, on the other hand, those who developed recurrent disease are more likely to have pulmonary comorbidities diagnosed over a lifetime (for example, in DS patients with recurrent PH following a diagnosis of Persistent Pulmonary Hypertension of the Newborn (PPHN), OSA are frequently diagnosed in 73% of cases, intermittent hypoxia in 67%, chronic lung disease in 47% and chronic aspiration in 47% of cases). In addition, uncorrected septal defects allow systemic to pulmonary circulation shunting, leading to PH [31].

#### 4.2.2. Ear Nose Throat (ENT) Involvement

Upper airways anomalies are already discussed above.

Dysphagia is another frequent comorbidity in DS children, because of some anatomical features, like craniofacial and structural abnormalities, and physiological characteristics, such as neuromotor coordination impairments, which can cause feeding problems and swallowing dysfunction [32]. The occurrence of pulmonary aspiration may lead to pneumonia, chronic respiratory symptoms and sometimes pulmonary hypertension, which, as already stated, can be in turn a cause of RRI. It is difficult to exclude silent aspiration on clinical feeding evaluations and chest radiographs may be normal too. It is therefore important to perform swallowing studies, such as videofluoroscopic swallow study, and eventually to address these patients to an ENT specialist for a fibreoptic endoscopic evaluation of swallowing [33,34,35].

#### 4.2.3. Gastrointestinal Involvement

Gastrointestinal abnormalities are frequently reported in DS patients, with a prevalence of 3–13% of both functional and structural congenital malformations. Among them, duodenal stenosis or atresia plays a prominent role but also Hirschsprung disease, anal stenosis and oesophageal atresia/trachea–oesophageal fistula are more frequent in DS children. The latter can directly affect the respiratory system, leading to cough, bronchitis but also pneumonia due to recurrent aspiration [2].

Children with DS are more likely to present Gastroesophageal Reflux (GER). This makes them more prone to be hospitalized because of respiratory illness and therefore an underlying diagnosis of GER should always be taken into account in case of recurrent pneumonia, chronic cough or wheeze [36]. The presence of recurrent wheeze can sometimes mislead the clinicians towards an improper diagnosis of asthma, while viral infection-related—recurrent wheezing indeed occurs more frequently in children with Down Syndrome; asthma, usually triggered by Th2 inflammation, can rarely be confirmed in those patients. Recurrent wheeze could be related to other factors like GER, which can therefore remain untreated [37].

#### 4.2.4. Musculoskeletal Involvement and Obesity

Musculoskeletal complications, although less frequently reported, are almost universally present in children with DS.

The presence of scoliosis should always be considered, since almost 5% of DS patients has either clinical or radiological signs of it. In particular, patients who underwent early thoracotomies from congenital heart surgery need to be carefully assessed, given the risk of secondary thoracic curves [38]. In this particular subset of patients, the management of early onset scoliosis can be challenging, considering that spinal deformities can impact the development of pulmonary alveoli but, on the other hand, an early surgical approach can result in a restrictive lung disease [39].

Hypotonia and joint hyperlaxity may lead to a delayed acquisition of motor milestones. The literature reports that children with Down Syndrome are usually able to walk without aid at 23 months, while for the general paediatric population it is possible around 13 months [38,40]. Inflammatory arthritis, hip and knee instability and other balance impairments can also result in a lower level of physical activity, which is known to be associated with a higher frequency of respiratory tract infections [41].

DS is also associated with a higher prevalence of overweight and obesity [29], which are known factors impacting lung function and volume. In particular, in obese and overweight children a significant decrease of FEV1/FVC and FEF25-75 was observed [42] but also the pro-inflammatory effect of adipose tissue on airways (adipose tissue driven inflammation) plays an important role [43]. Since weight gaining is not inevitable, clinicians should provide early guidance regarding healthy dietary practices and physical activity.

#### 4.2.5. Other Organs and Systems Involvement

As already stated before, DS patients can be affected by a wide spectrum of congenital malformations, which can impact their health and represent an indirect cause for Recurrent Respiratory tract Infections.

Lastly, patients with Down Syndrome are more often hospitalised due to neurological, endocrinological, haematological and other complications. This exposes them to an increased risk of hospital-acquired respiratory infections, even if more difficult to precisely estimate.

### 4.3. Immunologic Impairments

The immune system in DS is extensively dysregulated, with both innate and adaptive systems being affected. This substantial impairment results in an increased risk of infections, a risen infections’ severity with poorer clinical outcomes and often in a longer period of hospitalisation [11].

#### 4.3.1. Innate Immunity

Innate immunity is the first fast-acting and non-specific defence against pathogens and involves anatomical barriers, chemical defences and effector cells such as mast cells, macrophages, neutrophils, dendritic cells and NK cells.

Regarding neutrophils, the first studies on the immune function in DS patients identified a significantly impaired chemotaxis [44] and decreased phagocytic activity [45]. The literature review also points out a reduction in CD11b expression (a receptor involved in neutrophil’s activation and migration toward the site of infection) at baseline in DS children, with, on the other hand, a significantly greater increase post-endotoxin stimulation compared to controls; this hyper-responsiveness is thought to be potentially responsible for negative inflammatory consequences [46].

Monocytes also appear to be impaired in chemotaxis. In addition, although reduced in their total count, there is an increased percentage of non-classical or CD14dimCD16+ sub-populations, which has been associated to chronic inflammation, sepsis and malignancies [47,48]. Furthermore, TLR2, a pathogen receptor implicated in Gram-positive infections and chronic inflammation, seems to be increasingly expressed on neutrophils and monocytes in DS patients. Higher TLR2 expression has also been reported in patients with rheumatoid arthritis, coeliac disease and autoimmune thyroiditis (all conditions that are more frequent in DS patients), suggesting a potential etiological role in these autoimmune diseases [49].

Evidence regarding NK cells is conflicting: some studies reported an increased number of NK cells (apparently supporting the theory of precocious ageing of immune system in Down Syndrome since older people normally have higher percentages of NK cells) but there is still lack of global consensus in the literature. Additionally, their functioning, rather than deficient, appears to be abnormal, in some cases over-responding to stimulation (e.g., IFNγ), while sometimes showing an impaired response (e.g., IL2) [48,50].

The complement pathway is also abnormal. Complement Factor H, whose function is to inhibit C3 to C3b conversion, appears to be downregulated in Down Syndrome, resulting in a pro-inflammatory effect [51]. On the other hand, chronic inflammation can result in a secondary hypocomplementemia due to factors consumption.

To prevent chronic inflammation and autoimmunity and, on the other hand, ensure an appropriate response to infections, a well-regulated cytokines production is also required. DS patients present impairment in both pro-inflammatory and anti-inflammatory pathways: TNF-α, IL-1β and IFN-γ are significantly increased, and many human and animal models have demonstrated how this is associated to poorer outcomes in case of sepsis; concurrently, IL-10 and IL-1ra are also increased and this could contribute to the higher rate of respiratory tract infections [48].

Further studies are needed to clarify the overall effect of each specific molecule, but current data and clinical observation seem indicate a global immune dysregulation rather than a simple immunodeficiency, whose consequences can be toward both a hyper-activation (chronic inflammation, auto-immunity, worse outcome in case of sepsis) and a lower efficacy (higher rate of infections, especially but not solely involving the respiratory tract) of the immune system and response.

#### 4.3.2. Adaptive Immunity

Adaptive immunity is the slower but more accurate response which specifically targets the pathogen and is able to “remember” the germ encountered. Components of adaptive immunity are T and B lymphocytes. In DS patients, T lymphocytes both CD4+ and CD8+ are initially reduced in number due to the absence of normal lymphocyte expansion in the first year of life [52]. Although a gradual increase in T cells count over the years is documented, an impaired response to antigens stimulation, such as phytohemagglutinin, has been reported [53]. Moreover, the thymus gland, which is the core of T-cell development and immune tolerance, has been proved to be smaller in size and hypocellular with a reduced count of mature thymocytes and regulatory T cells.

A reduced number of B lymphocytes is documented in infancy and adult DS patients, lacking B cells the ability to increase in number over the years. Particularly dysfunctional is the defect in B cell differentiation with a decrease in switched memory B cells, crucial in immunisations’ response. Lastly, regarding immunoglobulins (Ig), conflicting evidence is reported in the literature with most DS subjects showing adequate Ig levels [52,54]. However, a suboptimal immune response to vaccination is frequently assessed in DS patients [55,56] resulting in severe outcomes such as hospitalizations and Critical Care Unit admissions due to vaccine preventable diseases. A possible waning over time of adaptive immune response has been stated as responsible [57,58], claiming an increased attention in public health campaigns for this particular subset of patients.

## 5. Prevention

Children with DS should be fully vaccinated, in accordance with their national schedule for immunization. The influenza and pneumococcal vaccines should also be strongly considered in children with DS because of their higher risk of respiratory tract infections and more severe course. As already stated, in consideration of the possible suboptimal immune response to vaccination, blood tests can be performed to check if there has been an adequate response and if there is otherwise necessity for an additional booster dose. Multiple factors are involved in the vaccination response that can therefore vary (e.g., it is usually decreased when influenza or tetanus vaccines are administered but it can be normal whereas it is pneumococcal or HBV vaccines) [53]. Given that DS children produce less specific switched memory B cells than their siblings after primary vaccination while they seem to respond comparably to a booster dose of vaccine, it could be appropriate to approve tailored vaccination programs for the DS population [59].

When the criteria are met, children with DS should receive RSV prophylaxis with palivizumab [36]. Some countries, like Japan since 2013, have already extended the use of palivizumab to all children with Down Syndrome (and not only in case of prematurity, congenital heart disease or other listed comorbidities). A large retrospective survey showed that the cumulative RSV hospitalisation rate could be reduced in DS patients without risk factors like prematurity or CHD, while no significant change was identified in the severity of RSV infection, in terms of days of hospitalisation, use for and duration of oxygen therapy. These findings surely need to be confirmed with longer, rigorous prospective clinical trials, also in consideration that RSV seasons vary by year, and a cost-effective analysis is mandatory [60].

The new monoclonal antibody nirsevimab is currently under approval in Italy as well as in many other countries but preliminary studies have certified its efficacy in protecting infants against hospitalization for RSV-associated LRTs and against very severe RSV-associated respiratory infections, both in healthy late-preterm and term infants [61,62]. Preliminary data also support the application of this therapy in children with congenital heart or lung diseases. DS children may benefit of an extended application of this therapy because of an increased risk of severe bronchiolitis [63,64].

A potential role in preventing respiratory tract infections in DS children has been suggested for immunomodulatory molecules such as pidotimod, with additionally confirmatory studies required [65].

## 6. Conclusions

This review highlights the well-known increased risk of respiratory tract infections in DS patients; they account for more than 40% of hospital admissions, representing the most common cause of admission in DS patients and the second cause of mortality (after CHD) [29]. In order to prevent that, considering the high prevalence and the variety of congenital malformations in DS children, a multidisciplinary approach, as well as an accurate and periodical screening program for comorbidities, is mandatory. However, some conditions, such as tracheomalacia and other lower airways malformations, are more challenging to be diagnosed. Particularly, in case of recurrent pulmonary infections, there are several potential etiological factors that should be considered, often requiring second- or even third-level assessments. It is indeed important to consider that more comorbidities could be present at the same time with a consequent higher risk of severe course or fatality.

## Figures and Tables

**Table 1:** Principal malformations involved in RRI in DS patients.

System Involved	Malformation
Airways malformations	Upper airways malformations
	Laryngomalacia
	Subglottic stenosis
	Tracheomalacia
	Bronchomalacia
	Tracheal bronchus
	Obstructive Sleep Apnoea
Cardiovascular involvement	Congenital Heart Diseases
	PPHN
	Pulmonary Hypertension
ENT involvement	Dysphagia
Gastrointestinal involvement	Gastroesophageal Reflux
Other systems involved	Musculoskeletal
	Overweight—Obesity
	Other causes for hospitalisation
Immunologic impairment	Innate immunity
	Adaptive immunity

## Data Availability

No new data were created or analyzed in this study. Data sharing is not applicable to this article.
